# Cloning, Expression Analysis, 20-Hydroxyecdysone Induction, and RNA Interference Study of Autophagy-Related Gene 8 from *Heortia vitessoides* Moore

**DOI:** 10.3390/insects11040245

**Published:** 2020-04-15

**Authors:** Zhixing Li, Zihao Lyu, Qingya Ye, Jie Cheng, Chunyan Wang, Tong Lin

**Affiliations:** College of Forestry and Landscape Architecture, South China Agricultural University, Guangzhou 510642, China; 20183137117@stu.scau.edu.cn (Z.L.); lvzihao@stu.scau.edu.cn (Z.L.); yeqingya@stu.scau.edu.cn (Q.Y.); cj@stu.scau.edu.cn (J.C.); wang_chunyan2018@163.com (C.W.)

**Keywords:** autophagy, autophagy-related gene 8, RNA interference, 20-hydroxyecdysone, starvation, *Heortia vitessoides* Moore

## Abstract

Autophagy is a highly conserved and regulated process in eukaryotic cells and remodels cytoplasm, recovers essential nutrients, and disposes of unwanted cytoplasmic components. Autophagy-related gene (ATG) 8, identified in *Heortia vitessoides* Moore, which is an oligophagous pest of *Aquilaria sinensis* (Lour.), was characterized (*HvATG8*). Multiple sequence alignment showed that *HvATG8* possesses highly conserved domain structures. Stage- and tissue-specific expressions indicated that *HvATG8* is highly expressed in prepupal, pupal, and adult stages and in the midgut of larvae and abdomen of adults. Lack of function of *HvATG8* by RNA interference resulted in a significant decrease in survival rate and an increase in abnormal or nonviable phenotypes in *H*. *vitessoides*. Transition rate from larval to pupal stages was 33.0% and from pupal to adult stages was 15.0% after injection. Reduction of ATG8 expression reduced survival of *H*. *vitessoides*. Therefore, *HvATG8* possibly plays a key role in normal growth stage of *H*. *vitessoides*. *HvATG8* suppression downregulates *HvATG3* expression, suggesting that the two genes are interconnected. Further, *HvATG8* expression increased by 20-hydroxyecdysone treatment, starvation, and extreme temperature exposure. Starvation also altered expression of other ATGs in *H*. *vitessoide*. This study may be used to guide research on molecular mechanisms of autophagy in insects.

## 1. Introduction

*Aquilaria sinensis* (Lour.) Gilg (*Malvales*: *Thymelaeaceae*) is not only a traditional and precious natural spice, but it is also the only medicinal plant that can produce aloes [1,2]. The tree is found throughout southern China, including in Guangdong, Fujian, and Yunnan provinces; Hong Kong; and Southeast Asia [3]. *A*. *sinensis* is the principal source of Chinese agarwood, a resinous heartwood formed in response to fungal infection, a moth whose leaf-eating larvae use *A*. *sinensis* as a sole food source [3]. The moth annually produces up to seven- to eight-generational alternation in southern China, and its larvae entirely defoliate *A*. *sinensis*, causing serious financial losses [4,5,6]. Control methods such as avermectin, sex pheromone, and trichlorfon administration have been used for infestations [7]. However, the moth has developed resistance to these common insecticides. Many reports on the biological and ecological characteristics of the moth are available [8], but few reports have focused on molecular control of *H*. *vitessoides* [8].

Autophagy is a self-digesting system of cells, which plays a pivotal role in maintaining the stability of the intracellular environment [9]. Three different types have been mentioned to date: microautophagy, chaperone-mediated autophagy, and macroautophagy [10]. Microautophagy means autonomous depression and deformation of lysosomal membranes or vacuolar membranes, which envelop and engulf part of the cytoplasm. An autophagy vesicle has a double-layer membrane structure, and its outer membrane can be fused with the lysosomal membrane. After autophagy vesicles enter the lysosome, the inner membrane is rapidly degraded, and vesicle contents are released, which is degraded by the hydrolytic enzyme; this allows cells to reuse substrates. In chaperone-mediated autophagy, receptors on the surface of lysosomal membranes selectively bind to soluble cytosolic proteins and regulate their transport to lysosomes. The formed chaperone–substrate complex is bound to the receptor of the lysosomal membrane, and the substrate is transported by the secondary chaperone protein in the cavity [11]. Macroautophagy, a degradation process focused on eliminating macromolecules and organelles, is the main pathway of autophagy in eukaryotic cells, which communicates a highly conducted self-degradation process that is started as an adaptive response under adverse conditions, for instance, nutritional removal. Usually, autophagy is referred to as macroautophagy. It is an intracellular process that degrades proteins in cytoplasmic components and organelles using lysosomes in eukaryote organisms ranging from yeast to mammals [11]. Autophagy has been extensively studied in yeast, and the functional analysis of 42 autophagy-related genes (ATGs) was carried out. Eighteen core ATG proteins are key regulators of different sections of autophagy [12,13]. 

Autophagy is induced during intracellular structural remodeling to respond to environmental stresses, to encapsulate and digest nonfunctional cell components into lysosomes, and to recycle nutrients. These activities are critical for the maintenance of normal cell metabolism and function [12,13]. Under adequate nutrition environment, a central inhibitor of autophagy is the target of rapamycin complex 1 (TORC1), which integrates various extracellular signals such as nutrition, energy, growth factors, and amino acids. It plays a crucial role in cell growth and apoptosis, and information from multiple upstream signals is collected and autophagosome initiation is inhibited by phosphorylation unc-51-like autophagy-activating kinase 1 (ULK1). Highly reactive TORC1 can restrain the occurrence of autophagy [14]. Under insufficient nutrition conditions, in addition to inhibiting TORC1 activity, the energy sensor AMPK can bind to ULK1 and directly phosphorylate, thus inducing autophagosome initiation. For example, autophagy is induced during starvation in mouse organs such as the liver, muscle, and pancreas [15]. It also occurs in adipocytes in the fat body of larvae of *Drosophila melanogaster* [16,17,18], which is the counterpart of the mammalian liver. The main cell types in insect fat bodies are adipocytes, whose morphological features include lipid droplets, glycogen rosettes, and protein granules. In most cellular organisms, autophagy is involved in a greater variety of physiological processes, such as development, immunity, stress protection, and tumor suppression. Autophagy can be induced by starvation and is a survival response for coping with unusual conditions [19], e.g., in the tick *Haemaphysalis longicornis* [20] and the greater wax moth *Galleria mellonella* [21]. New research shows that 20-hydroxyecdysone (20E) can also induce autophagy [22]. Furthermore, autophagy is involved in cell development processes including embryogenesis and metamorphosis in *D*. *melanogaster*, mammals, and *Caenorhabditis elegans* [23]. Recent studies indicate that autophagy mainly has three stages: induction of autophagy, autophagosome formation, and autophagosome–lysosome fusion [24]. 

Autophagy-related protein 8 is one of two ubiquitin-like proteins involved in autophagy that communicates protein lipidation with phosphatidylethanolamine (PE) by the ubiquitin-activating enzyme E1-like enzyme ATG7 and ubiquitin-conjugating enzyme E2-like protein ATG3 [25,26]. The number of ATG8 available decides the size of the autophagosome formed. The fact that ATG8 is still located in autophagosomes makes this protein a reliable marker for induction and progression of autophagy. Because ATG8 is the only ATG protein remaining on the autophagosome membrane after autophagosome formation, the formation of the ATG8–PE complex is the rate-limiting step in the occurrence of autophagy. Therefore, the study of ATG8 in many ATG genes has biological significance. These molecular mechanisms have been studied extensively in mammals, but regulation of autophagy in insect cells has rarely been reported, particularly regarding post-translational modification. ATG8 has been partially characterized in insect species including *G*. *mellonella* [21], *Bombyx mori* [27], *Aedes aegypti* [28], and *Spodoptera litura* [29]. The characterization of these ATG8s has revealed that ATG8 is a key factor in insects’ development. Nevertheless, the literature contains no reports on the characterization of ATG8 in the Lepidopteran insect *H*. *vitessoides* (*HvATG8*). 

In this study, a *HvATG8* homolog was identified, cloned, and characterized by silencing *HvATG8* with RNAi as well as immunofluorescence targeting. Then, the transcription pattern was defined for responses to treatment with 20E, starvation, and extreme temperatures. Through the analysis of expression patterns and functions, the ATG8 status of *H*. *vitessoides* under unfavorable environments and the morphological changes and survival rates caused by silenced ATG8 were clearly demonstrated, further confirming the importance of studying ATG8; this provides theoretical support for further exploration of its biological functions and application of ATG8 insecticides.

## 2. Materials and Methods 

### 2.1. Insects

*H*. *vitessoides* individuals were fed in a laboratory at a temperature of 26 °C and relative humidity of 70% ± 2%. Insects were sustained under a 14:10 h diurnal cycle. The larvae were reared with *A*. *sinensisata* leaves to promote into adults. We transferred mature larvae into plastic containers with sand at a relative humidity of 12% for later research. 

### 2.2. Sample Preparation

The larval stage of this insect has 5 instar stages; the entire larval stage takes 16–18 days for completion: approximately 10 days of the pupal stage and 2–4 days of the adult stage. There are slight differences depending on the environment. For stage-specific expression profiling of the target gene, 90 first-instar larvae (a biological repeat includes 30 larvae), 6 of the third- to fifth-instar larvae (a biological repeat includes 2 larvae), 45 instar larvae (a biological repeat includes 15 larvae), 6 pupae, and 6 adults (a biological repeat includes 2 larvae) were selected. Selecting a representative stage, it was observed whether the target gene has an important role in metamorphosis and whether it is expressed throughout the stage. All of the abovementioned steps were repeated thrice. 

One-day-old fifth-instar (L5D1) larvae were dissected into the head, epidermis, fat body, foregut, midgut, and hindgut; 1-day-old adults were dissected into the head, antenna of male and female, thorax, abdomen, leg, and wing. All samples were quickly frozen in liquid nitrogen and stored at −80 °C for total RNA extraction and cDNA synthesis.

Concerning starvation treatment, 50 stochastically selected L5D1 larvae for each experimental group were deprived of food for 96 h and sampled at 12 h intervals for analysis.

### 2.3. Sequence Verification and Phylogenetic Analysis

Through the *H*. *vitessoides* transcriptome (SRX3035102; [4]), the ATG8 sequence was acquired. cDNA sequences of the ATG8 open reading frames were acquired using ORF finders (http://www.ncbi.nlm.nih.gov/gorf/gorf.html). Corresponding pairs of *HvATG8*-specific primers were designed by Primer Premier 5.0 (Premier Biosoft International, Palo Alto, CA, USA) to verify the sequences. PCR amplification conditions were as follows: 95 °C for 5 min, 34 cycles of 30 s at 95 °C, 58 °C for 30 s, 72 °C for 2 min, and 72 °C for 10 min. The PCR product was gel purified and then roped into a pClone007 simple vector (TSINGKE Bio, Guangzhou, China), translated into *Escherichia coli* DH5α competent cells (Takara Bio, Otsu, Japan), and sequenced to confirm the target gene. The ATG8 amino acid sequences of other insect species were retrieved from the National Center for Biotechnology Information database. Sequence editing and multiple sequence alignment were performed with Clustal Omega (http://www.ebi.ac.uk/Tools/msa/clustalo/) and DNAMAN 6.0. Physicochemical properties of the ATG8 amino acid sequence were predicted using the ExPASy online server (http://web.expasy.org/compute_pi/). A neighbor-joining tree was generated using MEGA-X [30] and ClustalX with the neighbor-joining method, then bootstrap support was determined based on 1000 bootstrap replicates.

### 2.4. RNA Extraction and cDNA Synthesis

Total RNA was isolated from *H. vitessoides* larvae and adults by using the E.Z.N.A. Total RNA Kit II (OMEGA Biotec, Norcross, GA, USA) according to the manufacturer’s instructions. RNA concentrations were detected using a NanoDrop™ 2000 spectrophotometer (NanoDropProducts, Wilmington, DE). First-strand cDNA was synthesized with 2 µg total RNA from each sample using the Prime Script RT reagent kit with a gDNA eraser (Takara BioInc., Japan) and then directly stored at −20 °C for later use.

### 2.5. Primer Design and Quantitative Real-Time Polymerase Chain Reaction (RT-qPCR)

Primers were designed by Primer Premier 5.0 (Premier Biosoft International) and synthesized by TSINGKE Biotech Co., Ltd. All primer sequences are shown as supplementary data in Table S1. Total RNA was extracted from the samples, measured, and used for synthesis of first-strand cDNA. The relative transcript levels of target genes were measured using RT-qPCR to check specificity and amplification efficiency of primers and were normalized to the reference gene α-tubulin (GenBank accession: MG132200; [31]). Aseptic ultra-pure water was treated as negative control. The reaction system used 20 μL cDNA template, 10 μL TB Green Premix ExTaq, 0.4 μL forward primer, 0.4 μL reverse primer, and 7.2 μL double-distilled H_2_O. Amplification conditions were as follows: initial denaturation at 95 °C for 5 min, 40 cycles at 95 °C for 10 s, 60 °C for 20 s, and cooling at 40 °C for 30 s. Negative controls were nontemplate reactions (cDNA replaced with diethyl-pyrocarbonate water). For RT-qPCR, the LightCycler® Real-Time PCR System was used. Three biological replicates and three technical replicates were used in RT-qPCR. The 2^−ΔΔCt^ method was used in calculating the quantities of ATG8 mRNA [32].

### 2.6. dsRNA Preparation and Injection

The reagents for RNAi experiments in dsRNA synthesis were acquired from the T7 RiboMAX™ Express RNAi System (Promega, Madison, WI, USA). To obtain the DNA template, primers with the T7 polymerase promoter sequence were designed for routine PCR. DNA templates for *HvATG8* and green fluorescent protein were used with T7 RNA polymerase to form ds*ATG8* (314 bp) and ds*GFP* (pGWB5, 400 bp) fragments. The DNA template was removed, followed by dsRNA annealing and single-stranded RNA (ssRNA) removal through nuclease digestion. Then, dsRNA was purified on the basis of the manufacturer’s purification protocol (Promega). After purification, dsRNA was dissolved in nuclease-free water, quantified using a NanoDrop 2000 spectrophotometer (Thermo Fisher Scientific, Waltham, MA, USA), and, in order to ensure the purity and integrity, 1.5% agarose gel electrophoresis was used. 

The ds*HvATG8* solution was diluted to a concentration of 3 μg/μL for injection, and each larva was injected with 1 μL of this solution. L5D1 insects were injected at the lateral internodal membrane by FemtoJet (Eppendorf, Hamburg, Germany). The same concentration and dosage of ds*GFP* and DEPC were injected into controls [33]. At least 50 larvae were included in each group to confirm survival, pupation, and eclosion rates. Phenotypic changes were recorded during the experiments. Three biological repeats per treatment were used to evaluate the efficiency of RNAi. *HvATG8* transcript expression levels were recorded every 12 h until the 96^th^ h by RT-qPCR.

### 2.7. Phenotype Observation and Analysis

The treated insects should be checked discreetly for phenotypic changes. Individuals that did not react to brush touching within 1 min were considered dead. Surviving individuals were retained and continued to be monitored. 

### 2.8. 20E Preparation

We purchased 20E from Shanghai Yuanye Biotechnology Co., Ltd. (Shanghai, China), then diluted to 10 mg/ml with dimethyl sulfoxide and stored at −20 °C. The stock solution was diluted to a concentration of 1 μg/μL with 1× phosphate-buffered saline (PBS), and the injection volume was 1 μL. There were 30 larvae in each treatment that were injected between the membrane of the seventh and eighth segments. Each trial included three biological replicates and three technical replicates. Insects were collected in 1× PBS at 12, 24, 36, 48, and 60 h after injection and then reserved at −80 °C.

### 2.9. Starvation Treatment

L5D1 larvae were divided into three groups (n = 50) and deprived of food for 96 h. Larvae were collected at 12 h intervals and stored at −80 °C until further use.

### 2.10. Extreme Temperature Treatment

L5D1 larvae were selected in each group (n = 30). Larvae were exposed for 2 h to temperatures of 0 °C, 3 °C, 5 °C, 10 °C, 26 °C, 30 °C, 35 °C, 37 °C, and 40 °C. 

### 2.11. Statistical Analysis

Excel (Microsoft) was used for primary statistical analysis. Experimental data are rendered as mean ± standard error through three independent replicates. We used GraphPad Prism 8 to create charts. Differences were considered statistically significant with *p* < 0.05. One-way analysis of variance and Tukey’s test or two-way analysis of variance and Students’ t-test with SPSS 18. 

## 3. Results

### 3.1. Sequence Analysis of Hvatg8 and Phylogenetic Analysis

Based on *H*. *vitessoides* transcriptome data, ATG8 sequences were identified through keyword search and online comparison and designated as *HvATG8* (GenBank accession number: MN788362). The sequence has an open reading frame of 357 bp length and encodes 118 amino acids, and the theoretical molecular mass is 29.54 kDa with a predicted isoelectric point of 5.27 (Figure S1). 

Sequence alignment and BLAST search indicated that the amino acid sequence of HvATG8 was highly similar to that of Atg8 in other insects such as *B*. *mori BmATG8* (75.71%; NP 001040244), *S*. *litura SlATG8* (77.07%; JX183217), *Spodoptera*. *frugiperda SfATG8* (84.79%; Sf2M09420-5-1), *Trichoplusia ni TnATG8* (76.33%; JX183216), *Helicoverpa armigera HaATG8* (76.34%; JQ739159), and *Ostrinia furnacalis OfATG8* (85.84%; AYU75107.1). Amino acid sequences of ATG8 among insects appear highly conserved (Figure S2). 

**Figure 1:**
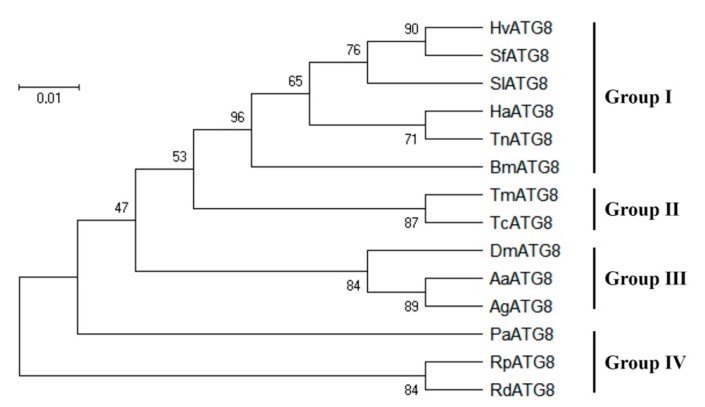
Phylogenetic analysis of *HvATG8*. The predicted amino acid sequence of *HvATG8* together with 13 selected Atg8 members was aligned, and the phylogenetic tree was constructed using MEGA X. GenBank accession numbers are: SfATG8, *S*. *frugiperda* (SPODOBASE: Sf2M09420-5-1); SlATG8, *S*. *litura* (JX183217); HaATG8, *H*. *armigera* (JQ739159); TnATG8, *T*. *ni* (JX183216); BmATG8, *B*. *mori* (NP_001040244.1); TmATG8, *Tenebrio molitor* (KM676434.1); TcATG8, *Tribolium castaneum* (XP_973073.1; DmATG8, *Drosophila melanogaster* (NM_167245.2); AaATG8, *Aedes aegypti* (AY736002.1); AgATG8, *Anopheles gambiae* (AY736002.1); PaATG8, *Periplaneta* *americana* (AB856588); RpATG8, *Riptortus pedestris* (BAN20392.1); and RdATG8, *Recilia dorsalis* (ATV91621.1).

After the above alignment, we found that the sequence of *HvATG8* is highly similar to that of other insects. We used MEGA-X to construct a phylogenetic tree to show the relationship in the middle of insects belonging to *Lepidoptera* (Group I), *Coleoptera* (Group II), *Diptera* (Group III), *Blattaria*, *Hemiptera*, and *Homoptera* (Group IV). *HvATG8* was most closely related to ATG8 in *S*. *frugiperda* with strong bootstrap support (Figure 1).

### 3.2. Stage-Specific and Tissue-Specific Expression Patterns of HvATG8

**Figure 2:**
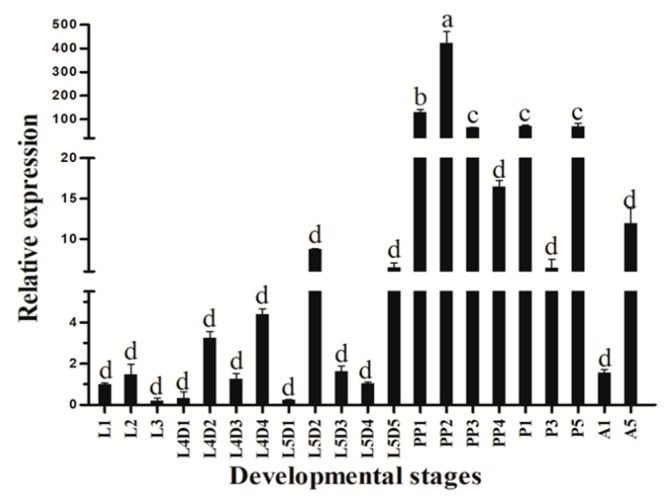
Relative expression levels of HvATG8 at different stages: L1–L3, first- to third-instar larvae; L4D1–L4D4, 1-day-old fourth-instar larvae to 4-day-old fourth-instar larvae; L5D1–L5D5, 1-day-old fifth-instar larvae to 5-day-old fifth-instar larvae; PP1–PP4, 1-day-old to 4-day-old prepupae; P1, 1-day-old pupae; P3, 3-day-old pupae; P5, 5-day-old pupae; A1, 1-day-old adults; and A5, 5-day-old adults. Its expression levels at each developmental stage were normalized relative to that at L1. Error bars represent mean ± standard error of three biological replicates. Different letters above error bars indicate significant differences (*p*<0.05) based on one-way analysis of variance and Tukey’s test.

*HvATG8* expression was determined at developmental stages in larvae and adults. *HvATG8* was detected at all stages. From the chart, expressions were lower at L1, L2, L3, L4, L5, and A1 than at other stages but were higher at prepupal and pupal stages and A5 (Figure 2). 

**Figure 3:**
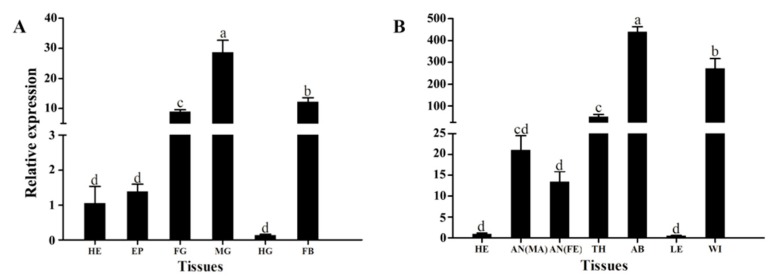
Relative expression levels of HvATG8 in different tissues of larvae and adults (tissues of 40 fifth-instar larvae were dissected). (**A**) Relative expression in larval tissues: HE, head; EP, epidermis; FG, foregut; MG, midgut; HG, hindgut; and FB, fat body. (**B**) Relative expression in adult tissues: HE, head; AN, antenna; TH, thorax; AB, abdomen; LE, leg; and WI, wing. Internal reference gene was α-Tubulin. Error bars represent mean ± standard error of three biological replicates. Different letters above error bars mean significant differences (*p* < 0.05), based on one-way analysis of variance (ANOVA) followed by Tukey’s test.

Moreover, among tissues, expressions of *HvATG8* transcripts were the highest in the midgut of larvae (Figure 3A) and highest in the abdomen of adults (Figure 3B). 

### 3.3. Silencing of HvATG8 by RNAi 

**Figure 4:**
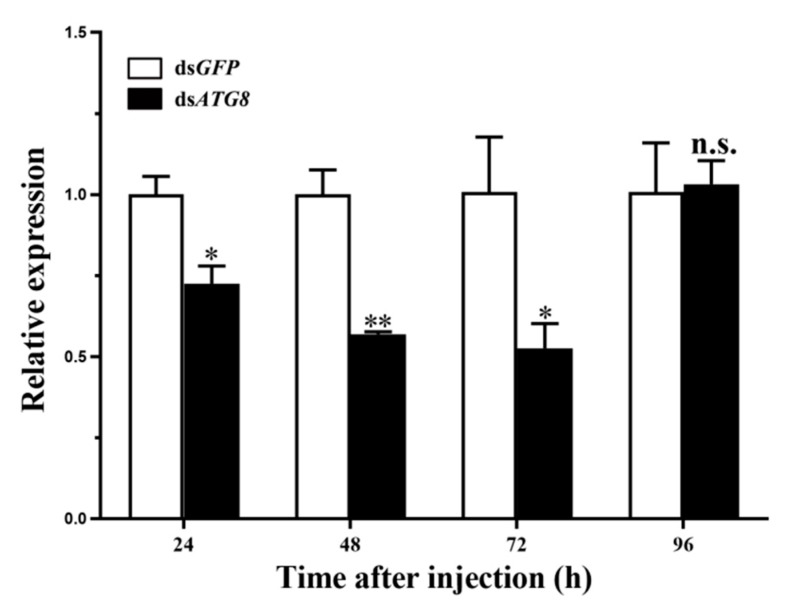
Changes in the messenger RNA level after treatment with specific RNA interference. Relative transcript levels of *HvATG8* in L5D1 larvae after injection with *dsHvATG8* at a concentration of 3.0 μg/μL for 24, 48, 72, and 96 h. The sample size was 150 larvae that were divided into three biological replicates. Error bars represent mean ± standard error of three biological replicates. * *p* < 0.05, ** *p* < 0.01, n.s. not significant. According to two-way analysis of variance (ANOVA) followed by u-test.

The total RNA was extracted from the whole body of dsRNA-injected larvae, and decrease in *HvATG8* expression via RNAi was detected using RT-qPCR. The expression level of *HvATG8* decreased relatively, with its highest expression levels being lower than ds*GFP* expression levels. After injection into L5D1 larvae, whole-body *HvATG8* level was the lowest at 72 h after injection. At this point, *HvATG8* mRNA levels were approximately 50% of the control level (Figure 4). This exposure time was used in subsequent work. 

### 3.4. Gene Expression of ATGs after ATG8 Silencing

**Figure 5:**
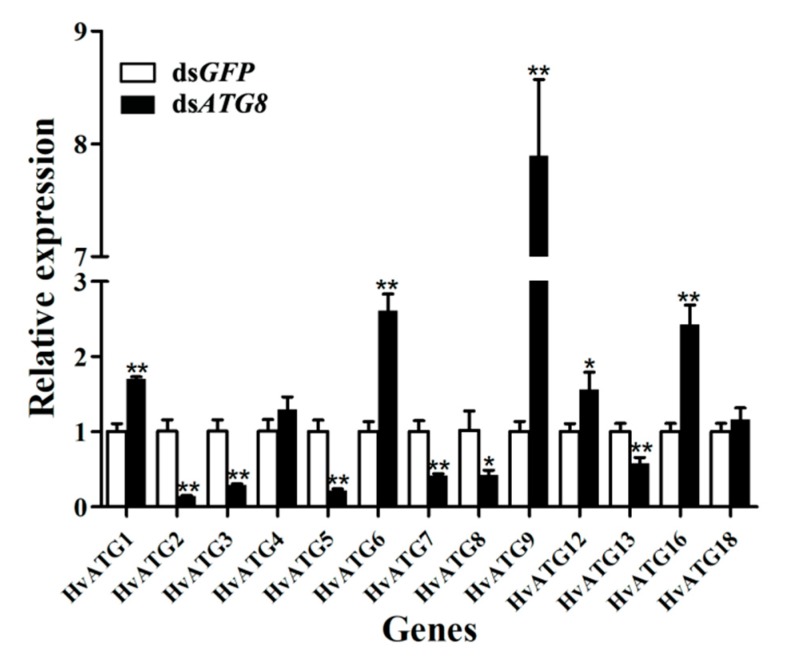
Expression levels of 13 *HvATGs* at 72 h after *HvATG8* silencing (n = 50 larvae in each biological replicate). Error bars represent mean ± standard error of three biological replicates. * *p* < 0.05, ** *p* < 0.01, via two-way analysis of variance followed by Students’ t -test.

After *HvATG8* silencing for 72 h, the relative gene expression levels of other ATGs were detected by RT-qPCR. While the transcript levels of *HvATG2*, *HvATG3*, *HvATG5*, *HvATG7*, and *HvATG13* significantly decreased, those of *HvATG1*, *HvATG6*, *HvATG9*, *HvATG12*, and *HvATG16* significantly increased, and those of *HvATG4* and *HvATG18* insignificantly increased (Figure 5). 

### 3.5. Phenotype Analysis and Survival Assay after RNAi 

**Figure 6:**
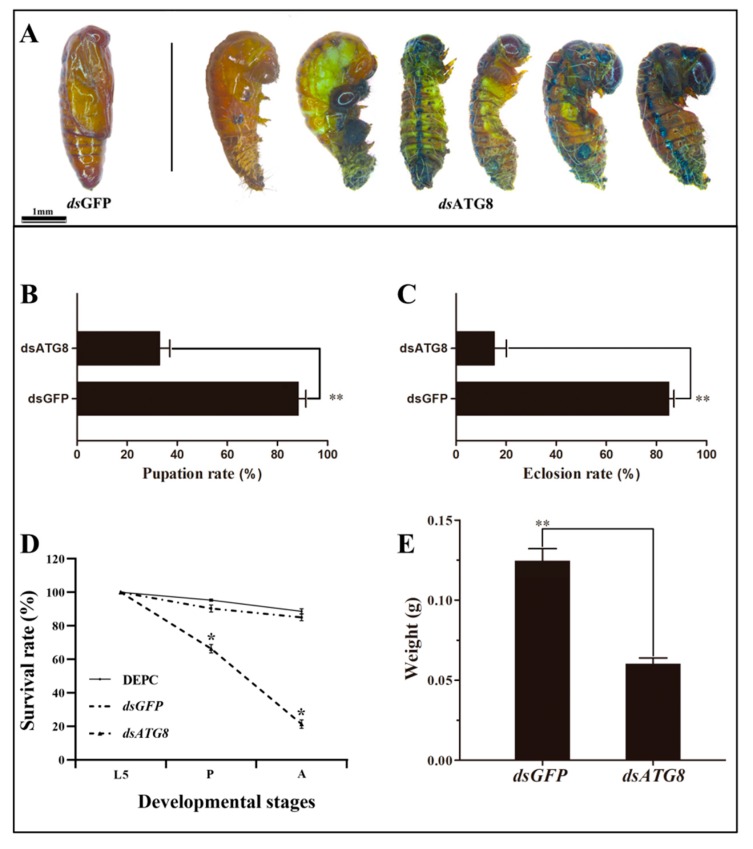
Effects of *HvATG8* RNAi on larval-to-pupal and pupal-to-adult transition rate. (A) Lethal phenotypes caused by *HvATG8* silencing. (B) Pupation rate after ds*HvATG8* and ds*GFP* injections. (C) Eclosion rate after ds*HvATG8* and ds*GFP* injections. (D) Survival changes in *H*. *vitessoides* after ds*HvATG8* injections at 96 h after injections (* *p* < 0.05, using Kaplan–Meier survival analysis with the log-rank test). (E) Larval weight at 72 h after ds*HvATG8* and ds*GFP* injections. These data were recorded separately based on a sample size of 150 larvae. Error bars represent mean ± standard error of three biological replicates. * *p* < 0.05, ** *p* < 0.01 based on Students’ t-test.

The insects injected with *HvATG8*-specific dsRNA separately exhibited abnormal and nonviable phenotypes. No phenotypic abnormalities were observed in ds*GFP* and DEPC (control insects; Figure 6A). After injection, larval–pupal transition rate was 33.0% (Figure 6B) and pupal–adult transition rate was only 15.0% in controls (Figure 6C). *T*. *castaneum* [33], *H*. *armigera* [34], and other species showed similar results after gene silencing. Despite a significant decrease in pupation and eclosion rates, the survival rate of L5D1 injected with ds*HvATG8* was 66.5% during the larval to pupal transition and 22.3% during the pupal to adult transition after injection. These rates were significantly lower than those in the control groups (Figure 6D). Average larval weight at 72 h was 71% of that in controls (Figure 6E). Mortality rate increased sharply in larvae starved after injection. All larvae died within 48 h.

### 3.6. Expression of HvATG8 after 20E Injection

**Figure 7:**
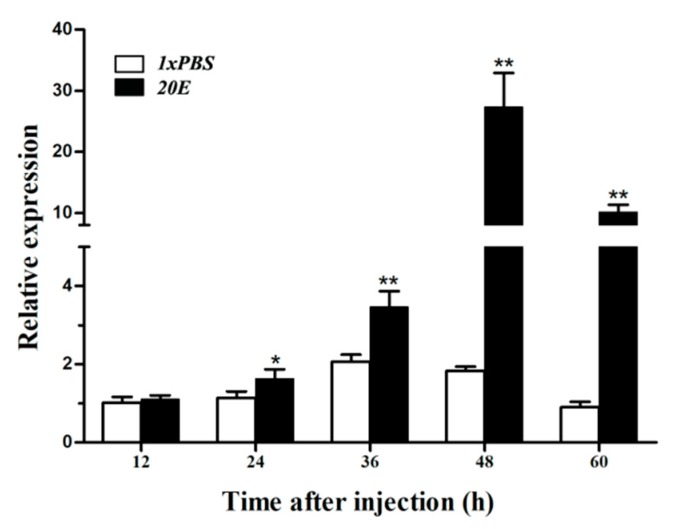
Expression patterns of *HvATG8* after 20E treatment (n = 30 larvae in each biological replicate). The 20E treatment results were compared relative to those after injection with 1× PBS (control). Error bars mean ± standard error of three biological replicates. Two-way analysis of variance followed by Students’ t-test was used. * *p* < 0.05, ** *p* < 0.01.

L5D1 larvae were treated with 20E and subjected to RT-qPCR. *HvATG8* mRNA transcript levels showed higher expression at 48 and 60 h after injection, with peak expression at 48 h after injection; this expression was almost 15-fold higher than that in controls at the same time point. After 60 h, mRNA transcript levels decreased, although expression remained obviously higher than in controls (Figure 7).

### 3.7. Starvation Treatment

**Figure 8:**
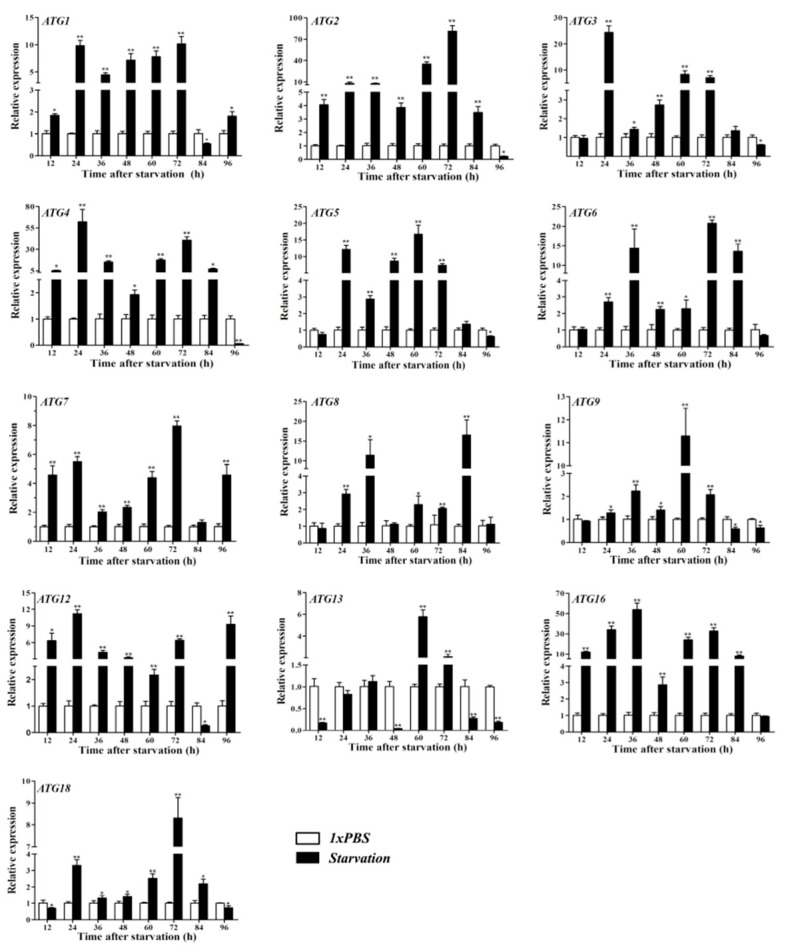
Expression patterns of *HvATGs* after 96 h of starvation (n = 50 larvae in each biological replicate). Expression levels of *HvATG1*, *HvATG2*, *HvATG3*, *HvATG4*, *HvATG5*, *HvATG6*, *HvATG7*, *HvATG8*, *HvATG9*, *HvATG12*, *HvATG13*, *HvATG16*, and *HvATG18*. Error bars mean ± standard error of three biological replicates calculated by two-way analysis of variance and Students’ t-test. * *p* < 0.05, ** *p* < 0.01.

During starvation, mRNA expression was measured at eight time intervals ranging from 12 to 96 h. A majority of treatment groups showed higher expression than in corresponding controls (Figure 8). Relative expression was the highest after 84 h of starvation.

### 3.8. Extreme Temperature Treatment 

**Figure 9:**
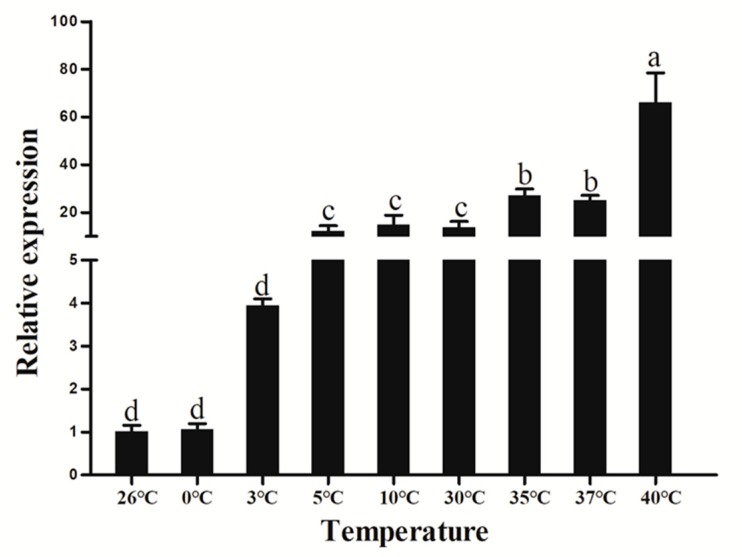
Relative expression levels of *HvATG8* across different temperatures (n = 30 larvae in each biological replicate). Expression levels at each temperature were normalized relative to that at 26 °C. Error bars represent mean ± standard error of three biological repeats. Different letters above error bars indicate significant differences (*p* < 0.05), according to one-way analysis of variance (ANOVA) followed by Tukey’s test.

ATG8 activity was detected in L5D1 larvae when in a series of temperatures, i.e., 0 °C, 3 °C, 5 °C, 10 °C, 30 °C, 35 °C, 37 °C, and 40 °C. *HvATG8* expression markedly increased relative to that in controls (26 °C) when larvae were exposed to high or low temperatures. Among all temperatures, the highest *HvATG8* expression level was detected at 40 °C, which was approximately 70 times that detected at 26 °C (Figure 9).

## 4. Discussion

Studies have shown that autophagy is an evolutionarily conservative pathway. When autophagy occurs, the material in the cell can be isolated in the double-membrane-bound autophagosome, and then enter the autophagosome–lysosome degradation [35,36]. After autophagosome formation, ATG8 remains the only ATG protein on the autophagosome membrane. ATG–PE level is an indicator of autophagic activity [37]. An ATG8 gene (*HvATG8*) from the transcriptome of adult *H*. *vitessoides* was identified in this study (Figure S1). Homologous comparison and phylogenetic tree analysis showed that *HvATG8* is highly homologous with *ATG8*s in other insect species such as *B*. *mori*, *D*. *melanogaster*, and *A*. *mellifera*. *HvATG8* has a higher homology with ATG8s in insects belonging to Group I (Figure 1) [38]. 

Expression patterns vary across developmental stages and among insect species, e.g., metamorphosis in fruit flies and dauer formation in nematodes [39]. In female *A*. *aegypti* mosquitoes, autophagy plays a pivotal role in maintaining the egg maturation cycle [40]. The developmental expression profile of *HvATG8* indicates that *HvATG8* is expressed at all stages of development and that its expression is higher at prepupal–adult stages but lower at larval stages (Figure 2). This expression pattern also appears in *Aedes albopictus, AaATG8* [41] and *Tenebrio molitor*, *TmATG8* [42]. This may be due to the fact that the prepupal–adult transition requires more material energy than other transitions, and more extensive autophagy occurs during this period than during other periods. This profile further reflects the profound restructuring necessary for the later stages of metamorphosis [36,43,44,45]. Autophagy is an essential part of insect development [46]. In *T*. *molitor*, *TmATG8* is relatively highly expressed in the midgut throughout development [47]. In the process of metamorphosis of *Drosophila*, larval tissues (midgut, salivary gland, and fat body) undergo autophagic degradation [42]. In *G*. *mellonella*, the expressions of *GmATG8* are detected in midgut, ovary, malpighian tubules, fat body, and silk gland, with the highest expression level in the midgut of larvae [21]. Tissue-specific expression revealed that *HvATG8* expression was the highest in the midgut of larvae (Figure 3). Autophagy may play a pivotal role in midgut remodeling in time of metamorphosis and is essential for midgut cell death in *D*. *melanogaster* [18]. The midgut is likely to participate in and control more of autophagy in *H*. *vitessoides*. In the present study, a variety of evidence shows that changes in the midgut of larvae during metamorphosis involve autophagy in Lepidoptera [46].

Proper dsRNA concentration is critical for RNAi experiments [9]. In *T*. *molitor*, ds*TmATG8* (1 μg/μL)-injected larvae experienced difficulties during ecdysis, including a higher death rate [47]. In the present study, through detection and comparison with a reference group, RNAi could be detected within 24 h, and the relative expression level of *HvATG8* declined to a minimum at 72 h before increasing (Figure 4). RNAi had the expected effect of reducing ATG8 expression; further, it indicated this to determine appropriate timing. Adverse effects occurred after ds*HvATG8* injection, particularly deformity and change of body colors (Figure 6A). The change of body colors may be due to the failure of the pupation process that the old stratum corneum of the body has not completely disappeared and a new epidermis has not been successfully formed. However, the state of the entire body is indeed a period of pupa. Reduced pupation rate (Figure 6B) and reduced emergence rate (Figure 6C) combined with changes in ATG8 level during the developmental period. When ATG8 was required to play a broad role, it was silenced. *H*. *vitessoides* could not obtain the necessary factors for growth and development, which would definitely hinder normal pupation and emergence as well as considerably increase the probability of deformity. Reduced survival (Figure 6D) suggests that RNAi-mediated silencing of ATGs impairs host ability to resist infection, and at the same time, insects are trapped in their old skins until they die due to the lower pupation and emergence rates because insects must undergo regular molting to continue to develop [47]. Such effects were not observed in larvae injected with DEPC and ds*GFP* as controls. Thus, *HvATG8* plays a key role in the development of *H*. *vitessoides*, and at the time of the use of RNAi for pest control, appropriate application timing needs to be determined. 

*Bombyx mori*, ATG3, and ATG8 have a special colocalization relationship [48]. A study of autophagy in yeast and other organisms found that ATG3 could act on ATG8 and eventually form the ATG8–PE complex [48]. Therefore, studying the interaction between them has important biological significance. In *B*. *mori*, *BmATG8* needs *BmATG3* for autophagy to occur [49], and ATG3 and ATG8 were likely to also be closely related in *H*. *vitessoides*. The relative expression level of *HvATG3* decreased when *HvATG8* was silenced (Figure 5). However, it remains unclear whether *HvATG8* silencing reduced *HvATG3* expression, but ATG8 and ATG3 interact to catalyze autophagy in *H*. *vitessoides*. In addition to *HvATG3*, the relative expression levels of other autophagy-related proteins showed significant changes after *HvATG8* silencing. Mutual promotion or restriction relationships between *HvATG8* and other autophagy-related proteins may occur. On this basis, we need to further elucidate the interactions between these genes and gene products in the future.

20E treatment is the most common method used to induce autophagy; during autophagy, its expression level increases significantly [50]. It activates the expression of 20E primary response genes such as E74, E75, and Br-C by binding to its receptor EcR-USP, thus inducing autophagy [51]. 20E can upregulate the expression of ATG8 in *Bombyx* [22] and *Drosophila* [22]. 20E could regulate the activity of ATG8 in many species. Based on this evidence, a functional connection between 20E and *HvATG8* was hypothesized, which needs to be examined. 20E injection led to increased *HvATG8* expression 48 h after treatment compared with in controls (Figure 7). 

In *Drosophila*, it has been found that EcR is decisive for 20E signaling to induce autophagy [52]. In addition, in *Bombyx*, 20E decreased TORC1 activity, upregulated ATG genes, and then induced autophagy [53,54]. ATG8 was quickly converted to ATG8–PE after 20E treatment, thereby inducing autophagy [55]. In *Drosophila*, 20E treatment induces autophagy by blocking TORC1 activity to induce autophagosome initiation [56]. Injection of 20Eblocked TORC1 activity and initiated autophagosome synthesis via ATG8 phosphorylation, and it upregulated ATG8 expression at the transcriptional level [22]. *HvATG8* expression can be induced by 20E. Thus, the expression of ATG8 in vivo may be regulated by 20E. It is not clear how autophagy promotes cell survival or death in different vitessoides larval tissues, and further research is warranted.

Starvation could stimulate ATG8 expression such as that seen in *H*. *longicornis* [20] and *G*. *mellonella* [21]. This phenomenon also occurs in *P*. *americana* [21]. The rapid increase in autophagy after starvation might be prompted by the exhaustion of amino acids by symbionts; in particular, glutamine and leucine are found in hemolymph because both amino acids are involved in TORC1 [57]. Autophagosomes or autophagic vacuoles also existed in digestive cells during starvation but not during feeding in ticks [58]. In *B*. *mori*, the expression level of *BmATG1* increased during starvation [35]. After 96 h of starvation, *H*. *vitessoides* demonstrated a period of high expression of autophagy-related proteins (Figure 8). Timing of expression varied across genes as starvation progressed, and autophagy was triggered. The underlying mechanism may be as follows: autophagy leads not only to the digestion of cytoplasmic components for recovering amino acids during amino acid deprivation [37] but also has an effect in the formation of protein granules in fat body during transformation. Starvation reduces the amount of protein granules and motivates the formation of autophagic compartments in trophocytes and the appearance of autophagic compartments in nidi and columnar cells. These observations indicate that starvation could stimulate autophagy [38]. Collectively, these data suggest that starvation plays a crucial role in inducing autophagy, confirming previously reported findings.

Studies have shown that temperature is often an important climatic factor affecting insect development [38]. Expressions of some insect genes have been studied at extreme temperatures, but no such studies have been conducted on ATG8. Extreme temperatures induced dramatic increases in *HvATG8* expression in fifth-instar larvae (Figure 9). The highest expression levels were observed at 40 °C. On one hand, ATG8 complexes are likely activated by phosphorylation and dephosphorylation in response to extreme temperatures. On the other hand, it may be that extreme temperatures inhibit TORC1 activity in the body and enhance the occurrence of autophagy. Notwithstanding, the present study suggests that extreme temperatures induce autophagy, with the mechanism of occurrence requiring further research 

## 5. Conclusions

The ATG8 sequence was successfully identified using the *H*. *vitessoides* cDNA library, and amino acid sequences of the gene product exhibited structural features conserved across different insect species. *HvATG8* was expressed at different stages and tissues in *H*. *vitessoides*. Specific analyses revealed that *HvATG8* was highly expressed at prepupal to adult stages and in the midgut of adults. From abnormal or nonviable phenotypes to decreased pupal weight and lower survival rate at the fifth-instar larval to adult stage, ds*ATG8* (3 μg/μL) injection caused *HvATG8* silencing with peak inhibition at 72 h. Decrease in *HvATG8* expression decreased *HvATG3* expression, and the determination of the underlying mechanism for this observation warrants further research. RT-qPCR showed that *HvATG8* expression could be caused by 20E. Its expression level is upregulated after starvation treatment, and ATGs in *H*. *vitessoides* are sensitive to starvation. Expression level also increased in response to extreme temperatures. Thus, extreme temperatures can trigger autophagy. In this study, RNAi-mediated targets were identified for pest control. 

## Supplementary Materials

The following are available online at https://www.mdpi.com/2075-4450/11/4/245/s1. Figure S1: sequencing of HvATG8; Figure S2: comparison of amino acid sequences of HvATG8 with Atg8 in other insects; Figure S3: some statistical results of two-way ANOVA; Table S1: PCR primers used in this study; Table S2: GenBank accession numbers.
