# Supplementary material for: Cloning, Expression Analysis, 20-Hydroxyecdysone Induction, and RNA Interference Study of Autophagy-Related Gene 8 from Heortia vitessoides Moore
Source: Insects. 2020 Apr 15;11(4):245. doi: 10.3390/insects11040245 (PMC7240406; doi:10.3390/insects11040245)
Supplement: Supplementary file 1 [file insects-11-00245-s001.pdf]

insects

MDPI

Article

Cloning, expression analysis, 20-hydroxyecdysone induction, and RNA interference study of autophagy-related gene 8 from *Heortia vitessoides* Moore

Zhixing Li, Zihao Lyu, Qingya Ye, Jie Cheng, Chunyan Wang and Tong Lin*

College of Forestry and Landscape Architecture, South China Agricultural University, Guangzhou,
* Correspondence: lintong@scau.edu.cn; Tel.: +86 020 85282217 (T.L.)

Supplementary Materials

```
TATTGGAATACAACACTGCTTCCTGCATTGTCATATCAGCTGATTGCGTCATTGGGTTTA     60

TAGACAATAACGCAAAAACAAAAAACAAGTCGTAGTATCCAGTTTTGCTATATTTTGATT     120

ATCTCGAAATTTTCAGTGCATTTTCATTCTCAAGATGAAATTCCAATATAAAGAGGAACAC    181
                                  M  K  F  Q  Y  K  E  E  H
TCGTTTGAAAAAAGGAAGACTGAGGGAGAAAAAATACGCAGGAAGTATCCGGACCGAGTT     241
 S  F  E  K  R  K  T  E  G  E  K  I  R  R  K  Y  P  D  R  V
CCAGTAATTGTTGAGAAAGCCCCGAAGGCTAGACTTGGAGACCTCGATAAGAAGAAGTAT     301
 P  V  I  V  E  K  A  P  K  A  R  L  G  D  L  D  K  K  K  Y
TTAGTGCCGTCTGATTTAACTGTTGGGCAGTTCTACTTCTTGATCAGAAAACGCATTCAC     361
 L  V  P  S  D  L  T  V  G  Q  F  Y  F  L  I  R  K  R  I  H
TTGCGGCCCGAAGATGCGCTGTTCTTCTTTGTCAACAATGTAATCCCTCCTACATCGGCC     421
 L  R  P  E  D  A  L  F  F  F  V  N  N  V  I  P  P  T  S  A
ACCATGGGCTCTTTGTACCAGGAACACCATGACGAAGATTTTTTCCTCTACATAGCATTC     481
 T  M  G  S  L  Y  Q  E  H  H  D  E  D  F  F  L  Y  I  A  F
TCCGACGAGAATGTTTATGGATATTTTTGAATTCCACGATTTCATCACCAGAATGTGAA      540
 S  D  E  N  V  Y  G  Y  F  *

ATTATATGATCATAATGGTGTAACTAGATTTAAGGCTTAGCTTAATGATTGATGTATTAT     600

GTTATAATGGTGATAATTATTTTTAATTTGCAATTCTACAAACAACTCTGTCAAATCACA     660

TTGTTAGAGTTTAACATCGGTTTAAATGAAAATATTGCATTGTTTTAATCTCTTTGTTTG     720

GTTTAGGAGTGTGATTATTTCTGTATCAAAATAAATATAAAAAGTACAAAAAATTGCGTC     780

TTTCTTTCATTCTAAAGGTATTAAAGACCTTATTTTATGTGCTAAAGTATGGTTCATATA     840

GTTCATTCTTATATTATGAGACAGGTAATGTCGTGAATTGTTCTATAAACCATCTACTTG     900

GTCTAATTTACACAATTAATAATTCGGAAAACAACATACATATTAGGGAAAATACTATTA     960

ATATAATATTTTACTCTGTATGAACAGAACATCATAATATTAACAGTAAATCATTCCTTA     1020

TACTTTAGATTTTATAGGTATTAATTCTATTCATGCG                            1057
```

Figure S1. Sequence analysis of HvATG8. Nucleotide sequence of the cDNA encoding Atg8 isolated from Heortia vitessoides and the deduced amino acid sequence .The initiation codon and the termination codon are marked with a red underlines.

```
HvATG8    MKFQYKEEHSFEKRKTEGEKIRRKYPDRVPVIVEKAPKARLGDLDKKKYLVPSDLTVGQF    60
SfATG8    MKFQYKEEHSFEKRKTEGEKIRRKYPDRVPVIVEKAPKARLGDLDKKKYLVPSDLTVGQF    60
OfATG8    MKFQYKEEHSFEKRKTEGEKIRRKYPDRVPVIVEKAPKARLGDLDKKKYLVPSDLTVGQF    60
SlATG8    MKFQYKEEHSFEKRKTEGEKIRRKYPDRVPVIVEKAPKARLGDLDKKKYLVPSDLTVGQF    60
HaATG8    MKFQYKEEHSFEKRKTEGEKIRRKYPDRVPVIVEKAPKARLGDLDKKKYLVPSDLTVGQF    60
TnATG8    MKFQYKEEHSFEKRKTEGEKIRRKYPDRVPVIVEKAPKARLGDLDKKKYLVPSDLTVGQF    60
BmATG8    MKFQYKEEHSFEKRKAEGEKIRRKYPDRVPVIVEKAPKARLGDLDKKKYLVPSDLTVGQF    60
AaATG8    MKFQYKEEHPFEKRKAEGDKIRRKYPERVPVIVEKAPKARIGDLDKKEYLVPSDLTVGQF    60
TmATG8    MKFQYKEEHPFEKRKSEGEKIRRKYPDRVPVIVEKAPRARIGDLDKKKYLVPSDLTVGQF    60
PaATG8    MKFQYKEEHPFEKRKAEGEKIRRKYPDRVPVIVEKAPKARIGDLDKKKYLVPSDLTVGQF    60
          ********* *****:**:*******:**********:**:******:************

HvATG8    YFLIRKRIHLRPEDALFRFV-NVIPPTSATMGSLYQEHHDEDFFLYIAFSDENVYGYF--    118
SfATG8    YFLIRKRIHLRPEDALFRFVNNVIPPTSATMGSLYQEHHDEDFFLYIAFSDENVYGYY--    118
OfATG8    YFLIRKRIHLRPEDALFRFVNNVIPPTSATMGSLYQEHHDEDFFLYIAFSDENVYGSF--    117
SlATG8    YFLIRKRIHLRPEDALFRFVNNVIPPTSATMGSLYQEHHDEDFFLYIAFSDENVYGY---    117
HaATG8    YFLIRKRIHLRPEDALFRFVNNVIPPTSATMGSLYQEHHDEDFFLYIAFSDENVYGY---    117
TnATG8    YFLIRKRIHLRPEDALFRFVNNVIPPTSATMGSLYQEHHDEDFFLYIAFSDENVYGY---    117
BmATG8    YFLIRKRIHLRPEDALFRFVNNVIPPTSATMGSLYQEHHDEDFFLYIAFSDENVYGN---    117
AaATG8    YFLIRKRIHLRPEDALFRFVNNVIPPTSATMGSLYSEHHEEDYFLYIAYSDENVYGNK--    118
TmATG8    YFLIRKRIHLRPEDALFRFVNNVIPPTSATMGSLYQEHHEEDFFLYIAYSDENVYGGDEL    120
PaATG8    YFLIRKRIHLRPEDALFRFVNNVIPPTSATMGSLYQEHHEEDFFLYIAYSDENVYGQ---    117
          ******************** **************.***:**:*****:*******
```

Figure S2. Comparison of amino acid sequences of HvATG8 with Atg8 in other insects. SfATG8, *Spodoptera frugiperda* (SPODOBASE: Sf2M09420-5-1); OfATG8, *Ostrinia furnacalis* (AYU75107.1)SlATG8, *Spodoptera litura* (JX183217); HaATG8, *Helicoverpa armigera* (JQ739159); TnATG8, *Trichoplusia ni* (JX183216); BmATG8, *Bombyx mori* (NP_001040244.1); TmATG8, *Tenebrio molitor* (KM676434.1); AaATG8, *Aedes aegypti* (AY736002.1) and PaATG8, *Periplaneta americana* (AB856588). Tyr49 and Leu50, which are required for the activation of the lipidated form of Atg8 by Atg7 and Atg3, are boxed in red. Phe77 and Phe 79, which are parts of the recognition site for Atg4, are boxed in pink (* indicate identical residues).

A **Test of Between-Subjects Effects**

Development Variable: Expression

Source	TypeIII Sum of Squares	df	Mean Square	F	Sig.
Corrected Model	1157.455[a]	5	231.491	7.243	0.000
intercept	768.239	1	768.239	24.038	0.000
species	404.772	1	404.772	12.665	0.002
Time	752.683	4	188.171	5.888	0.002
Error	767.029	24	31.960		
Total	2692.723	30			
Corrected Total	1924.484	29			

a. R Squared = 0.601（Adjusted R Squared = 0.518）

B **Test of Between-Subjects Effects**

Development Variable: Expression

Source	TypeIII Sum of Squares	df	Mean Square	F	Sig.
Corrected Model	0.749[a]	4	0.187	9.266	0.000
intercept	17.710	1	17.710	876.353	0.000
species	0.511	1	0.511	25.279	0.000
Time	0.238	3	0.079	3.928	0.024
Error	0.384	19	0.020		
Total	18.843	24			
Corrected Total	1.133	23			

a. R Squared = 0.661（Adjusted R Squared = 0.590）

Figure S3. Some statistical results of two-way ANOVA. A represents the result of RNA interference; B represents the result of 20-hydroxyecdysone treatment.

Table S1. PCR primers used in this study.

Primers	Primer sequence (5′–3′)
**For real-time PCR**	
HvATG1-F	CGCACACCTCAACCCTC
HvATG1-R	GTCCGCTTTGGCATCAT
HvATG2-F	CTCGACAGACGACACCCAC
HvATG2-R	GCTGACAAGTCCACACGCT
HvATG3-F	TTACCGCTTAGTTCCGT
HvATG3-R	CCTGTGTTGCCTTTCTC
HvATG4-F	CGAATCCCGCCTACTAT
HvATG4-R	TCCTTCAATGCCCTCTC
HvATG5-F	ATGTCAACTATGCAGAAGAAGG
HvATG5-R	CAGTGGAGTATCCACAGGAAG
HvATG6-F	CATTATGTGCCTCCTTTTCG
HvATG6-R	CCTCCTTGTCATCTTCCAGC
HvATG7-F	CCTCCTTGTCATCTTCCAGC
HvATG7-R	TGGAATGTGGTATGGGA
HvATG8-F	TGAAAAAAGGAAGACTGAGGG
HvATG8-R	CGGAGAATGCTATGTAGAGG
HvATG9-F	TGCCGTGTGTTGGAGAT
HvATG9-R	GGGCGAAGTGAAGCTAG
HvATG12-F	TCGGGTGGATTATGGAGTT
HvATG12-R	ATTTGGTCTGGAGAGGGTG
HvATG13-F	CGAGGATGGAGACGAAA
HvATG13-R	ATAGGCGACCGAAAAAC
HvATG16-F	CTGGTCTCCTGGCGTCTGT
HvATG16-R	CGCTTGCTTCCTCCTTTTT
HvATG18-F	AGCCCACAAAACCTTAC
HvATG18-R	GCACACCAGATCCAGAG
**For dsRNA**	
dsATG8-F	TAATACGACTCACTATAGGGAAAAGGAAGACTGAGGGAGA
dsATG8-R	TAATACGACTCACTATAGGGCGGAGAATGCTATGTAGAGG
dsGFP-F	TAATACGACTCACTATAGGGCAGTTCTTGTTGAATTAGATG
dsGFP-R	TAATACGACTCACTATAGGGTTTGGTTTGTCTCCCATGATG

F: forword primer; R: reverse primer.

Table S2. GenBank accession number in this study.

Gene	Genbank number
HvATG1	MN788355
HvATG2	MN788356
HvATG3	MN788357
HvATG4	MN788358
HvATG5	MN788359
HvATG6	MN788360

HvATG7	MN788361
HvATG8	MN788362
HvATG9	MN788363
HvATG12	MN788364
HvATG13	MN788365
HvATG16	MN788366
HvATG18	MN788367
α-tubulin	MG132200
